# Acute and subacute toxicity of an ethanolic extract of Melandrii Herba in Crl:CD sprague dawley rats and cytotoxicity of the extract in vitro

**DOI:** 10.1186/s12906-016-1342-3

**Published:** 2016-09-22

**Authors:** Eunsook Park, Mee-Young Lee, Chang-Seob Seo, Sae-Rom Yoo, Woo-Young Jeon, Hyeun-Kyoo Shin

**Affiliations:** K-herb Research Center, Korea Institute of Oriental Medicine, 1672 Yuseong-daero, Yuseong-gu, Daejeon, 34054 Republic of Korea

**Keywords:** Acute toxicity test, Cytotoxicity test, Herbal medicine, Melandrii herba, Subacute toxicity test, Rats

## Abstract

**Background:**

Melandrii Herba, a medicinal plant, has been used in Korea for treatment of bacterial and fungal infection. However, the safety and toxicity of Melandrii Herba have not yet been established. Therefore, we investigated the acute and subacute toxicity of an ethanolic extract of Melandrii Herba (MHEE) in Crl:CD Sprague Dawley rats and cytotoxicity of MHEE in vitro.

**Methods:**

To study acute toxicity, rats were treated with MHEE at single doses of 0, 500, 1000, and 2000 mg/kg administered by oral gavage, and body weight, clinical signs, and mortality were observed after dosing. To study subacute toxicity, rats were treated with MHEE at doses of 0, 500, 1000, and 2000 mg/kg administered once a day by gavage for 4 weeks. We measured clinical signs, mortality, gross pathological findings, body and organ weights, food consumption, serum biochemistry, and conducted hematology and urinalysis. The cytotoxicity of MHEE was assayed by measuring the viability of prostate cell lines including normal prostate stromal WPMY-1, normal prostate epithelial RWPE-1, and benign prostatic hyperplasia epithelial BPH-1 cells at various concentrations of MHEE in vitro.

**Results:**

Single oral doses of MHEE caused no significant difference in rat clinical signs, mortality, or body weight. The lethal dose of MHEE was considered to be >2000 mg/kg. Daily oral doses of MHEE for 4 weeks did not result in any significant changes in rat mortality, gross pathological findings, relative organ weights, food consumption, hematology, serum biochemistry, or urinalysis. At MHEE >1000 mg/kg/day, salivation was increased in both male and female rats. However, the salivation caused by the MHEE treatment was not accompanied by pathological changes in body weight or gross pathological findings, and we considered the salivation as a minor symptom. Therefore, no adverse effects were seen at 2000 mg/kg/day or less. MHEE showed no cytotoxic effects on either normal prostate or benign prostatic hyperplasia cell lines.

**Conclusions:**

Administration of MHEE in Crl:CD Spradgue Dawley rats is nontoxic and is safe for at least a month.

## Background

Herbal medicines have been used traditionally as therapeutic agents in Asia including Korea, China, and Japan. However, establishing information about the composition and toxicity of herbal medicines has often been overlooked. With increased interest in complementary and alternative medicine in modern society, scientific evidence for the efficacy of several herbal medicines for various disorders has been reported [1]. To assess the safety of the wide array of herbal medicine in clinical use, evaluation of the adverse effects of herbal medicines is ongoing [2].

Melandrii Herba, is the aboveground portion of fruiting *Melandryum firmum* Rohrbach. *M. firmum,* a biennial herbaceous plant, is widely distributed in Korea and Melandrii Herba has been used traditionally for the treatment of anuria, gonorrhea, breast cancer, and diseases of lactation [3]. Several compounds from Melandrii Herba have been isolated and identified including α-spinasterol, ursolic acid, flavonoids, and a saponin, which all exhibit pharmacological activities [4–6]. Components of Melandrii Herba have been found to possess anti-inflammatory and apoptotic activity in vitro and a protective effect in a rat model of benign prostatic hyperplasia (BPH) [5, 7, 8]*.* However, the toxicity or safety of Melandrii Herba administration has yet to be elucidated.

In this study, we investigated the acute toxicity of an ethanolic extract of Melandrii Herba (MHEE) to identify its approximate lethal dose and thereafter examined the subacute toxicity of daily doses of MHEE in Crl:CD Sprague Dawley rats for 4 weeks according to guidelines established by the Organization for Economic Cooperation and Development for the testing of chemicals in accordance with the current regulations for Good Laboratory Practice [9]. In addition, viability assays in prostate cell lines were conducted in vitro to determine the cytotoxicity of MHEE.

## Methods

### Plant materials

Melandrii Herba was purchased from Kwangmyungdang (Ulsan, Korea) in July 2014 and identified by Dr. Jung Hoon Kim, a pharmacognosist at the K-herb Research Center, Korea Institute of Oriental Medicine (KIOM; Daejeon, Korea). A voucher specimen (2014-GO-23) has been deposited at the K-herb Research Center, KIOM.

### Chemicals and reagents

Schaftoside, homoorientin, and isovitexin were purchased from Shanghai Sunny Biotech (Shanghai, China). Cytisoside was provided by Prof. Jong-Keun Son, College of Pharmacy, Yeungnam University, Korea. The purity of the four reference standards was at least 95 %. Methanol, acetonitrile, and water were HPLC-grade solvents and purchased from J.T. Baker (Phillipsburg, NJ). Trifluoroacetic acid was analytical reagent grade and purchased from Sigma-Aldrich (St Louis, MO).

### Preparation of MHEE

Dried Melandrii Herba (3.0 kg) was extracted with 70 % ethanol (30 L × 3) by ultrasonication for 60 min at room temperature. The extracted solution was filtered using filter paper, the ethanol was removed at 40 °C under vacuum (N-11; Eyela, Tokyo, Japan) and the concentrated extract lyophilized (PVTFD10RS; ilShinBioBase, Yangju, Korea). Extract lyophilized 345.5 g with yield (11.5 %) was obtained.

### High-performance liquid chromatography analysis of Melandrii Herba extract

High-performance liquid chromatography (HPLC) for the quantitative analysis of the four flavonoids, schaftoside, homoorientin, cytisoside, and isovitexin, was conducted on a Shimadzu Prominence LC-20A series system (Kyoto, Japan). This system consisted of a solvent delivery unit (LC-20AT), online degasser (DGU-20A3), column oven (CTO-20A), automatic sample injector (SIL-20 AC), and photodiode array detector (PDA, SPD-M20A). The data were acquired and processed using LC solution software (version 1.24; Shimadzu, Kyoto, Japan). Separation of the four flavonoids was achieved on a Gemini C18 column (250 mm × 4.6 mm, 5 μm, Phenomenex, Torrance, CA) maintained at 50 °C. The mobile phases consisted of 0.1 % (v/v) aqueous trifluoroacetic acid (A) and acetonitrile (B). The gradient flow was as follows: 5–10 % B for 0–10 min, 10–50 % B for 10–30 min, 50–100 % B for 30–40 min, 100 % B for 40–45 min, and 100–5 % B for 45–50 min. Re-equilibration time was 10 min. The flow rate was 1.0 mL/min and injection volume was 10 μL. For quantitative determination, 250 mg of lyophilized sample was dissolved in 50 mL of distilled 70 % methanol and then ultrasonicated for 20 min. After extraction, the solution was filtered through a 0.2 μm syringe filter (Pall Life Sciences, Ann Arbor, MI) before injection into the HPLC system.

### Animals

The animal studies were conducted according to the guidance of the Institutional Animal Care and Use Committee in the Korea Institute of Toxicology (KRICT) (accredited by AAALAC International, 1998) under the current Good Laboratory Practice regulations for nonclinical laboratory studies and approved by the Korea Institute of Oriental Medicine Institutional Animal Care and Use Committee (Daejeon, Korea). We obtained five (5)-week-old specific pathogen-free Crl:CD Sprague Dawley rats (*n* = 22/sex) from Orient Bio Co. (Seoul, Korea) and were used for the experiments after one week of acclimatization. The rats were housed in a room temperature at 22 ± 3 °C and a relative humidity of 50 ± 20 % with artificial lighting from 08:00 to 20:00 and 10–20 air changes per hour as described previously [10].

### Group assignment and treatment

Group assignment and treatment were as described previously [10]. Briefly, healthy male and female rats were assigned to four groups (*n* = 5/group) using a Path/Tox System (version 4.2.2; Xybion Medical Systems Corporation, Cedar Knolls, NJ). MHEE was dissolved in distilled water for oral administration (Choong-Wae Pharmaceutical, Korea).

To study its acute toxicity, MHEE was administered to the rats by oral gavage at doses of 0, 500, 1000, and 2000 mg/kg and the rats were monitored for mortality, body weight, and clinical signs for 14 days after a single dose. To study its subacute toxicity, MHEE was administered once daily by oral gavage at doses of 0, 500, 1000, and 2000 mg/kg for 4 weeks. MHEE was prepared freshly on each treatment day and the vehicle control group received an equal volume of distilled water. The daily dose (10 mL/kg body weight) of MHEE was calculated based on the most recently recorded body weights of individual rats.

### Necropsy

Gross postmortem examinations were performed after an overdose of anesthetic as described previously [11]. Absolute organ weights were measured and relative organ weights (organ-to-body weight ratios) were calculated for the following: brain, pituitary gland, adrenal gland, liver, spleen, kidneys, heart, thymus, lung, salivary gland, thyroids, testes, ovaries, epididymides, seminal vesicle, prostate, and uterus.

### Hematology, serum biochemistry, and urinalysis

All analyses were performed as described previously [10]. Briefly, blood samples for hematology were analyzed to determine red blood cell (RBC) count, white blood cell (WBC) count, differential WBC count, hemoglobin concentration (HGC), hematocrit (HCT), mean corpuscular volume (MCV), mean corpuscular hemoglobin (MCH), mean corpuscular hemoglobin concentration (MCHC), platelet (PLT), and reticulocyte (RET) count using an ADVIA120 Hematology System (Bayer).

Serum biochemistry analysis included measurement of alanine aminotransferase (ALT), aspartate aminotransferase (AST), alkaline phosphatase (ALP), gamma glutamyl transpeptidase (GGT), blood urea nitrogen (BUN), creatinine (CREA), creatine kinase (CK), glucose (GLU), total cholesterol (TCHO), albumin (ALB), albumin/globulin ratio (A/G), total protein (TP), triglyceride (TG), total bilirubin (TBIL), phospholipids (PL), sodium (Na), potassium (K), calcium (Ca), chloride (Cl), and inorganic phosphorus (IP).

Urinalysis included measurement of urine volume, glucose, ketone bodies (KET), bilirubin (BIL), specific gravity (SG), pH, and urobilinogen (URO).

### Cell culture and viability assay

Normal prostate stromal WPMY-1 cells and normal prostate epithelial RWPE-1 cells were obtained from the American Type Culture Collection (Rockville, MD), and benign prostatic hyperplasia epithelial BPH-1 cells were obtained from Creative Bioarray (Shirley, NY). WPMY-1 cells were maintained in Dulbecco’s Modified Eagle’s Medium (Gibco, Grand Island, NY) supplemented with 5 % fetal bovine serum (FBS; Gibco). RWPE-1 cells were maintained in keratinocyte Serum-Free Medium (Gibco) supplemented with 5 ng/mL of human epidermal growth factor and 0.05 mg/mL of bovine pituitary extract. BPH-1 cells were maintained in RPMI 1640 medium (Gibco) supplemented with 20 % FBS. The cells were cultured at 37 °C under an atmosphere of 5 % CO_2_.

Cytotoxicity was measured using a nonradioactive Cell Counting Kit-8 (CCK-8) (Dojindo, Japan) viability assay according to the manufacturer’s instructions. WPMY-1 and BPH-1 cells were cultured in phenol red-free medium containing 5 % charcoal-treated FBS. WPMY-1 (3 × 10^3^ cells/well), RWPE-1 (8 × 10^3^ cells/well), and BPH-1 (5 × 10^3^ cells/well) cells were seeded into 96-well plates and incubated with various concentrations (0, 3.13, 6.25, 12.5, 25, 50, or 100 μg/mL) of MHEE for 24 h. Cell viability was calculated as described previously [10].

### Statistical analyses

Statistical analyses were based on methods described previously [10]. Data collected during the study were examined for homogeneity of variance using Bartlett’s test. When Bartlett’s test indicated no significant deviation from homogeneity, a one-way analysis of variance (ANOVA) was performed at α = 0.05. When significant difference was noted using the ANOVA, a multiple comparison test (Dunnett’s *post hoc* test) was conducted to determine which pairs of groups were significantly different. When significant deviations from homogeneity of variance were noted, a nonparametric comparison (Kruskal–Wallis test) was conducted. The Dunn’s Rank Sum test was conducted to determine the specific pairs when a significant difference was observed in the Kruskal–Wallis test. *P* < 0.05 was considered significant. Values are presented as means ± SD.

## Results

### HPLC analysis of four flavonoids in Melandrii Herba

**Fig. 1:**
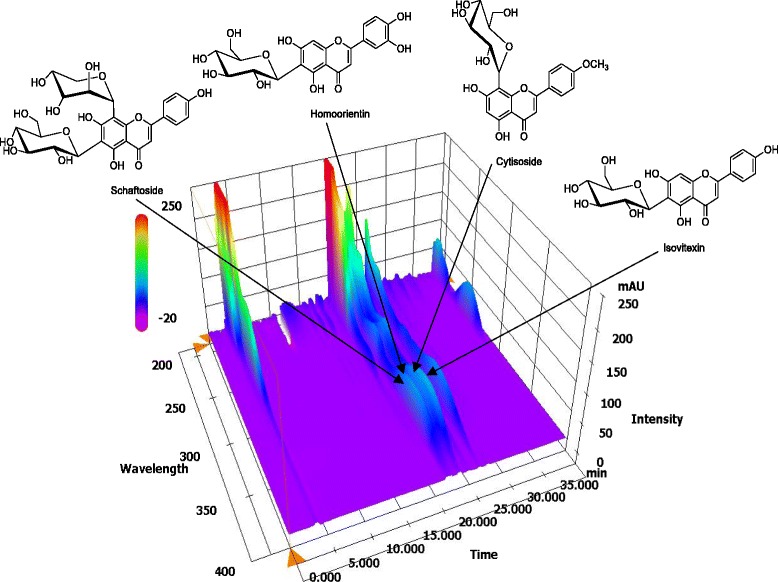
Three-dimensional HPLC-PDA chromatogram of Melandrii Herba ethanolic extract

In this study, an optimized HPLC-PDA method was applied to determine four flavonoids in the Melandrii Herba extract quantitatively. These compounds were eluted within 30 min with resolution ≥ 1.17. Representative typical three-dimensional chromatograms of Melandrii Herba extract obtained by HPLC-PDA are shown in Fig. 1. The retention time for schaftoside was 21.77 min, the time for homoorientin was 22.04 min, for cytisoside was 23.07 min, and for isovitexin was 23.57 min. The regression equation in the tested concentration range was y = 20818.78 × –3913.36 for schaftoside, y = 27432.50 × –5391.03 for homoorientin, y = 13601.45 × –5387.31 for cytisoside, and y = 39,667 × –7354.12 for isovitexin. The correlation coefficients of the four marker compounds were all 0.9999. The concentrations of the four flavonoids in the lyophilized Melandrii Herba extract were 4.78 mg/g for schaftoside, 2.37 mg/g for homoorientin, 11.57 mg/g for cytisoside, and 4.40 mg/g for isovitexin.

### Acute toxicity of MHEE

**Fig. 2:**
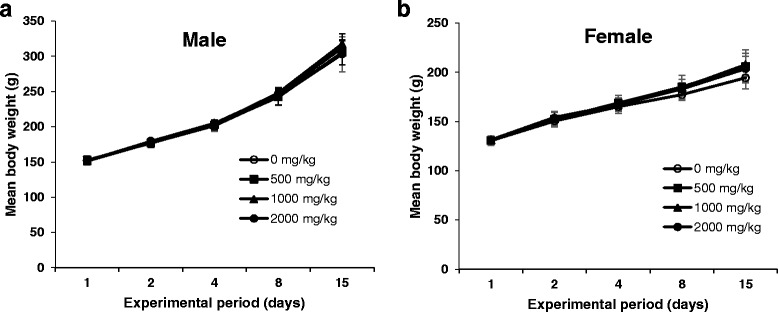
Mean of body weight change of male (**a**) and female (**b**) rats after a single dose of MHEE at 0 (○), 500 (■), 1000 (▲), and 2000 (●) mg/kg. Values are presented as means ± SD

To determine the appropriate dose of MHEE for testing of subacute toxicity, we first determined the acute toxicity of a single oral dose in male and female rats. Administration of MHEE at doses of 0, 500, 1000, and 2000 mg/kg resulted in no significant changes in body weights compared with the vehicle control group (Fig. 2). Moreover, no changes of clinical signs, mortality, or gross pathological findings were observed in MHEE-treated rats (data not shown). These data indicate that the lethal dose of MHEE in rats is >2000 mg/kg. Based on the results of this acute toxicity test, we next examined the subacute toxicity of MHEE in rats for 4 weeks.

### Clinical signs and mortality of rats treated with MHEE for 4 weeks

Salivation was observed in three male rats treated with MHEE at 1000 mg/kg/day, and all male rats treated with MHEE at 2000 mg/kg/day, and soft faeces were observed in just one male rat treated with MHEE at 2000 mg/kg/day (Table 1). Salivation was observed in one female rat treated with MHEE at 500 mg/kg/day, four female rats treated with MHEE at 1000 mg/kg/day, and all female rats treated with MHEE at 2000 mg/kg/day. No dead rats were seen in any group throughout the study period (data not shown).

**Table 1:** Clinical signs in rat treated orally with MHEE for 4 weeks

Group	Salivation^a^	Soft faeces^a^
Male rats		
0 mg/kg/day	0/5	0/5
500 mg/kg/day	0/5	0/5
1000 mg/kg/day	3/5	0/5
2000 mg/kg/day	5/5	1/5
Female rats		
0 mg/kg/day	0/5	0/5
500 mg/kg/day	1/5	0/5
1000 mg/kg/day	4/5	0/5
2000 mg/kg/day	5/5	0/5

^a^Number of animals with sign/Total number of animals observed

### Body weight and food consumption changes of rats treated with MHEE for 4 weeks

**Fig. 3:**
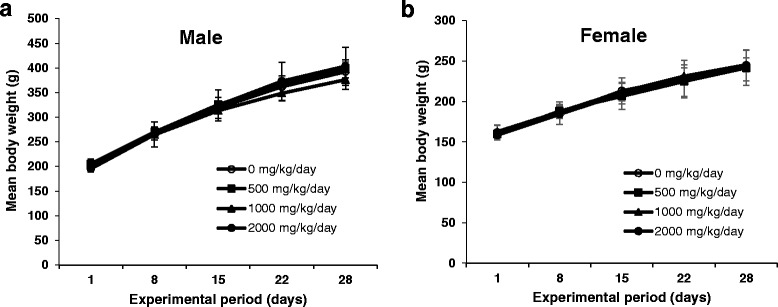
Mean of body weight change of male (**a**) and female (**b**) rats treated with MHEE at doses of 0 (○), 500 (■), 1000 (▲), and 2000 (●) mg/kg/day for 4 weeks. Values are presented as means ± SD

**Fig. 4:**
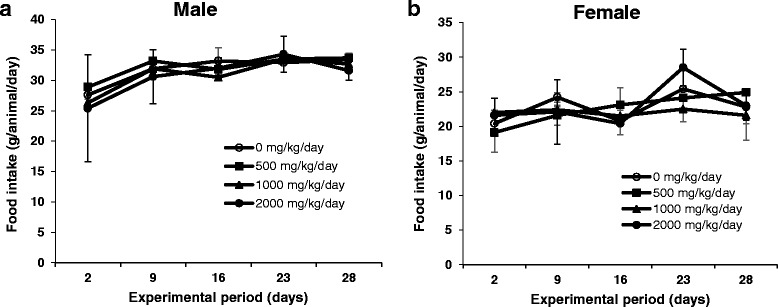
Food intake in male (**a**) and female (**b**) rats treated with MHEE at doses of 0 (○), 500 (■), 1000 (▲), and 2000 (●) mg/kg/day for 4 weeks. Values are presented as means ± SD

No significant differences in body weight (Fig. 3) or food consumption (Fig. 4) were found between rats in the vehicle control- and MHEE-treated groups, regardless of sex.

### Necropsy finding of rats treated with MHEE for 4 weeks

No treatment-related gross pathological changes were found in any rat at necropsy except for liver focus (*n* = 1; yellow color in median lobe) in a male rat treated with MHEE at 2000 mg/kg/day and a decrease in the size of the thyroid/parathyroid (*n* = 1) in a female rat treated with MHEE at 500 mg/kg/day (data not shown).

Relative organ weights (organ weight/fasted body weight) were calculated as shown in Table 2. The relative weight of the thyroid/parathyroid was increased significantly in male rats treated with MHEE at 500 mg/kg/day or more. The relative weight of the liver was increased significantly in female rats treated with MHEE at 2000 mg/kg/day. Except for the thyroid/parathyroid in male and the liver in female rats, relative weights of all other organs measured were not significantly different between rats in the vehicle control- and MHEE-treated groups.

**Table 2:** Relative organ weights (g) in rats treated with MHEE for 4 weeks

Dose (mg/kg/day)	0	500	1000	2000
Male rats				
Brain	0.540 ± 0.0392	0.532 ± 0.0381	0.560 ± 0.0204	0.531 ± 0.0577
Pituitary gland	0.033 ± 0.0003	0.003 ± 0.0002	0.003 ± 0.0004	0.003 ± 0.0004
Liver	3.207 ± 0.1068	3.104 ± 0.2479	3.280 ± 0.1780	3.422 ± 0.1196
Spleen	0.200 ± 0.0284	0.195 ± 0.0282	0.207 ± 0.0231	0.221 ± 0.0100
Heart	0.311 ± 0.0126	0.331 ± 0.0210	0.334 ± 0.0170	0.325 ± 0.0216
Thymus	0.150 ± 0.0148	0.155 ± 0.0241	0.143 ± 0.0200	0.179 ± 0.0294
Salivary glands	0.181 ± 0.0229	0.184 ± 0.0084	0.184 ± 0.0118	0.180 ± 0.0101
Seminal vesicle	0.321 ± 0.0326	0.349 ± 0.0404	0.351 ± 0.0575	0.303 ± 0.0457
Prostate	0.104 ± 0.0128	0.109 ± 0.0268	0.129 ± 0.0370	0.099 ± 0.0158
Kidneys	0.818 ± 0.0747	0.833 ± 0.0457	0.831 ± 0.0711	0.824 ± 0.0326
Adrenal glands	0.015 ± 0.0190	0.015 ± 0.0013	0.015 ± 0.0009	0.016 ± 0.0014
Testes	0.815 ± 0.0405	0.832 ± 0.0395	0.889 ± 0.0552	0.848 ± 0.1053
Epididymides	0.276 ± 0.0228	0.265 ± 0.0139	0.267 ± 0.0154	0.259 ± 0.0368
Lung	0.410 ± 0.0377	0.413 ± 0.0121	0.412 ± 0.0138	0.406 ± 0.0258
Thyroid/parathyroid	0.005 ± 0.0006	0.006 ± 0.0010*	0.006 ± 0.0006*	0.007 ± 0.0007**
Female rats				
Brain	0.813 ± 0.0508	0.849 ± 0.0871	0.809 ± 0.0386	0.840 ± 0.0616
Pituitary gland	0.006 ± 0.0006	0.006 ± 0.0007	0.006 ± 0.0004	0.006 ± 0.0007
Liver	3.138 ± 0.1330	3.278 ± 0.1348	3.33 ± 0.1695	3.497 ± 0.2084**
Spleen	0.219 ± 0.0495	0.233 ± 0.0307	0.222 ± 0.0183	0.234 ± 0.0250
Heart	0.388 ± 0.0533	0.382 ± 0.0153	0.357 ± 0.0074	0.371 ± 0.0156
Thymus	0.221 ± 0.0252	0.200 ± 0.0232	0.227 ± 0.0148	0.232 ± 0.0648
Salivary glands	0.181 ± 0.0112	0.197 ± 0.0108	0.188 ± 0.0125	0.201 ± 0.0131
Kidneys	0.859 ± 0.0590	0.917 ± 0.0565	0.837 ± 0.0945	0.905 ± 0.0303
Adrenal glands	0.031 ± 0.0025	0.033 ± 0.0032	0.030 ± 0.0036	0.033 ± 0.0043
Ovaries	0.042 ± 0.0049	0.041 ± 0.0041	0.037 ± 0.0067	0.045 ± 0.0061
Lung	0.543 ± 0.0140	0.544 ± 0.0306	0.537 ± 0.0332	0.522 ± 0.0406
Thyroid/parathyroid	0.008 ± 0.0006	0.007 ± 0.0013	0.008 ± 0.0014	0.008 ± 0.0011
Uterus/cervix	0.291 ± 0.1312	0.279 ± 0.0762	0.255 ± 0.1077	0.264 ± 0.0416

Values are presented as the mean ± SD

*, ** indicate a significant difference at *P* < 0.05 and *P* < 0.01, respectively, when compared with the vehicle control group

### Hematology, serum biochemistry, and urinalysis of rats treated with MHEE for 4 weeks

Hematological values are reported in Table 3. Levels of basophils were significantly reduced in male rats treated with MHEE at 1000 mg/kg/day or more, whereas the levels of monocytes were increased significantly in rats treated with 2000 mg/kg/day. However, there was no significant hematological change in female rats treated with MHEE at any concentration compared with vehicle-treated controls.

**Table 3:** Hematological values of rats treated with MHEE for 4 weeks

Dose (mg/kg/day)	0	500	1000	2000
Male rats				
WBC (10^3^/μL)	9.2 ± 3.08	8.4 ± 2.14	9.5 ± 1.02	9.6 ± 1.66
Reticulocytes (%)	2.8 ± 0.45	2.7 ± 0.31	2.4 ± 0.34	2.9 ± 0.64
Neutrophils (%)	11.6 ± 2.19	9.4 ± 3.11	8.0 ± 2.75	13.6 ± 9.17
Lymphocytes (%)	83.9 ± 2.51	86.1 ± 3.42	88.3 ± 2.64	80.2 ± 9.79
Eosinophils (%)	1.0 ± 0.44	0.9 ± 0.23	0.7 ± 0.17	0.7 ± 0.26
Monocytes (%)	1.8 ± 0.50	1.8 ± 0.42	1.7 ± 0.57	3.0 ± 0.48**
Basophils (%)	0.7 ± 0.14	0.6 ± 0.09	0.4 ± 0.13**	0.5 ± 0.04*
Large unstained cells (%)	1.1 ± 0.33	1.1 ± 0.27	0.9 ± 0.17	2.0 ± 0.75
RBC (10^6^/μL)	8.4 ± 0.24	8.2 ± 0.34	8.1 ± 0.25	8.0 ± 0.43
Hematoglobin (g/dL)	16.4 ± 0.63	16.4 ± 0.78	16.6 ± 0.63	16.5 ± 0.58
Hematocrit (%)	50.6 ± 1.32	50.4 ± 1.63	50.5 ± 0.98	50.2 ± 1.86
MCV (fL)	60.4 ± 1.32	61.5 ± 1.46	62.2 ± 1.24	63.1 ± 2.54
MCH (pg)	19.5 ± 0.50	20.1 ± 0.56	20.5 ± 0.61	20.7 ± 1.1
MCHC (g/dL)	32.3 ± 1.27	32.6 ± 0.55	33.0 ± 0.65	32.9 ± 0.80
Platelet (10^3^/μL)	1038.0 ± 114.18	980.0 ± 61.93	1015.8 ± 111.26	1014.6 ± 74.29
Female rats				
WBC (10^3^/μL)	5.9 ± 1.60	7.2 ± 1.06	8.6 ± 2.46	6.4 ± 1.06
Reticulocytes (%)	2.6 ± 0.52	2.6 ± 0.58	2.9 ± 0.25	2.7 ± 0.67
Neutrophils (%)	10.0 ± 1.90	14.8 ± 7.42	13.2 ± 6.83	7.8 ± 1.71
Lymphocytes (%)	85.7 ± 2.25	81.1 ± 7.82	83.3 ± 6.74	87.9 ± 1.95
Eosinophils (%)	1.0 ± 0.35	0.7 ± 0.17	0.9 ± 0.25	1.2 ± 0.64
Monocytes (%)	1.9 ± 0.41	1.7 ± 0.54	1.4 ± 0.11	1.7 ± 0.50
Basophils (%)	0.4 ± 0.19	0.4 ± 0.17	0.3 ± 0.16	0.4 ± 0.18
Large unstained cells (%)	1.0 ± 0.11	1.2 ± 0.18	1.0 ± 0.25	1.0 ± 0.21
RBC (10^6^/μL)	8.18 ± 0.42	8.3 ± 0.45	7.8 ± 0.34	8.1 ± 0.42
Hematoglobin (g/dL)	16.3 ± 0.87	16.1 ± 0.56	15.8 ± 0.43	16.0 ± 0.68
Hematocrit (%)	48.7 ± 2.36	48.6 ± 2.36	47.7 ± 1.84	47.6 ± 2.15
MCV (fL)	59.6 ± 0.80	58.8 ± 1.61	60.9 ± 0.92	59.2 ± 0.70
MCH (pg)	19.9 ± 0.28	19.5 ± 0.74	20.2 ± 0.66	19.8 ± 0.34
MCHC (g/dL)	33.4 ± 0.59	33.2 ± 0.99	33.1 ± 0.76	33.5 ± 0.41
Platelet (10^3^/μL)	1063.8 ± 47.47	1004.6 ± 104.58	1142.8 ± 121.17	1181.4 ± 182.15

Values are presented as the mean ± SD

*, ** indicate a significant difference at *P* < 0.05 and *P* < 0.01, respectively, when compared with the vehicle control group

*MCV* mean corpuscular volume, *MCH* mean corpuscular hemoglobin, *MCHC* mean corpuscular hemoglobin concentration, *PT* prothrombin time

Serum biochemical analysis is reported in Table 4. No significant changes in the levels of measured biochemical factors were found in male rats treated with MHEE compared with vehicle controls, whereas changed levels of some biochemical factors were observed in female rats treated with MHEE compared with vehicle controls. Levels of GLU, TP, and ALB were increased significantly in female rats treated with MHEE at 1000 mg/kg/day, and the level of Ca was increased after treatment with MHEE at 1000 mg/kg/day or more.

**Table 4:** Serum biochemical values of rats treated with MHEE for 4 weeks

Dose (mg/kg/day)	0	500	1000	2000
Male rats				
Glucose (mg/dL)	108.9 ± 23.89	118.7 ± 12.86	126.8 ± 10.29	118.3 ± 21.31
BUN (mg/dL)	14.6 ± 2.52	13.3 ± 0.64	12.9 ± 0.57	14.4 ± 2.56
Creatinine (mg/dL)	0.4 ± 0.03	0.4 ± 0.05	0.4 ± 0.05	0.4 ± 0.02
Total protein (g/dL)	6.2 ± 0.36	6.1 ± 0.17	6.1 ± 0.09	6.2 ± 0.21
Albumin (g/dL)	4.0 ± 0.18	4.0 ± 0.06	4.0 ± 0.05	4.0 ± 0.09
Albumin/globulin ratio	1.9 ± 0.15	1.9 ± 0.11	1.9 ± 0.09	1.8 ± 0.12
Total cholesterol (mg/dL)	55.2 ± 15.56	63.4 ± 7.30	55.2 ± 6.38	69.6 ± 14.94
Triglycerides (mg/dL)	21.3 ± 7.84	27.5 ± 8.48	18.0 ± 7.15	23.8 ± 7.19
Phospholipid (mg/dL)	86.2 ± 16.45	97.6 ± 10.01	84.8 ± 7.22	98.0 ± 14.09
AST (IU/L)	118.1 ± 9.24	116.8 ± 14.48	108.6 ± 16.78	110.7 ± 11.73
ALT (IU/L)	36.3 ± 3.50	29.2 ± 2.66	30.8 ± 3.75	32.3 ± 5.73
Total bilirubin (mg/dL)	0.1 ± 0.03	0.1 ± 0.01	0.1 ± 0.01	0.1 ± 0.03
ALP (IU/L)	714.6 ± 104.91	757.7 ± 109.10	557.0 ± 81.66	603.8 ± 124.47
Creatine kinase (IU/L)	497.2 ± 193.62	669.2 ± 362.80	473.2 ± 54.79	494.6 ± 119.26
Ca (mg/dL)	10.6 ± 0.25	10.8 ± 0.10	10.7 ± 0.18	11.1 ± 0.65
IP (mg/dL)	10.9 ± 0.42	11.1 ± 0.71	10.7 ± 0.64	11.9 ± 1.51
Na (mmol/L)	138.6 ± 0.55	139.2 ± 1.79	140.0 ± 1.58	140.0 ± 1.58
K (mmol/L)	9.0 ± 0.57	9.2 ± 1.13	8.0 ± 1.19	8.3 ± 0.97
Cl (mmol/L)	97.4 ± 1.14	97.6 ± 1.82	98.0 ± 1.58	96.6 ± 0.89
GGT (IU/L)	0.7 ± 0.29	0.7 ± 0.23	0.7 ± 0.18	0.7 ± 0.26
Female rats				
Glucose (mg/dL)	93.7 ± 18.39	111.1 ± 21.40	141.2 ± 27.07**	99.7 ± 17.64
BUN (mg/dL)	16.7 ± 3.18	18.8 ± 3.30	20.3 ± 3.62	19.0 ± 4.05
Creatinine (mg/dL)	0.4 ± 0.08	0.4 ± 0.04	0.5 ± 0.13	0.5 ± 0.09
Total protein (g/dL)	6.2 ± 0.37	6.6 ± 0.24	6.9 ± 0.24**	6.5 ± 0.17
Albumin (g/dL)	4.1 ± 0.17	4.3 ± 0.12	4.5 ± 0.26*	4.3 ± 0.10
Albumin/globulin ratio	2.1 ± 0.15	1.9 ± 0.10	1.9 ± 0.15	2.0 ± 0.03
Total cholesterol (mg/dL)	64.2 ± 10.47	67.6 ± 11.08	79.6 ± 11.61	59.6 ± 12.05
Triglycerides (mg/dL)	12.0 ± 3.98	12.8 ± 4.04	15.4 ± 5.87	7.1 ± 2.05
Phospholipid (mg/dL)	111.6 ± 20.60	124.0 ± 18.32	136.6 ± 18.54	103.2 ± 15.90
AST (IU/L)	118.3 ± 15.95	107.2 ± 16.03	120.8 ± 13.35	117.9 ± 13.45
ALT (IU/L)	24.2 ± 2.45	25.5 ± 3.60	26.9 ± 2.95	25.4 ± 4.51
Total bilirubin (mg/dL)	0.1 ± 0.02	0.1 ± 0.01	0.1 ± 0.02	0.1 ± 0.02
ALP (IU/L)	376.3 ± 106.87	400.8 ± 110.06	319.6 ± 57.75	370.5 ± 106.39
Creatine kinase (IU/L)	615.6 ± 155.68	417.2 ± 127.15	550.4 ± 185.10	621.2 ± 234.55
Ca (mg/dL)	10.3 ± 0.16	10.7 ± 0.31	10.9 ± 0.26**	10.7 ± 0.28*
IP (mg/dL)	9.3 ± 0.49	9.6 ± 0.98	9.2 ± 0.44	9.3 ± 0.75
Na (mmol/L)	138.2 ± 1.92	139.6 ± 1.34	139.6 ± 1.14	139.8 ± 0.84
K (mmol/L)	7.8 ± 0.37	7.7 ± 0.96	7.1 ± 0.86	7.4 ± 0.54
Cl (mmol/L)	99.2 ± 2.68	99.4 ± 1.67	98.8 ± 2.28	98.8 ± 1.92
GGT (IU/L)	1.2 ± 0.44	1.2 ± 0.19	1.2 ± 0.15	1.2 ± 0.30

Values are presented as the mean ± SD

*, ** indicate a significant difference at *P* < 0.05 and *P* < 0.01, respectively, when compared with the vehicle control group

*ALP* alkaline phosphatase, *AST* aspartate aminotransferase, *ALT* alanine aminotransferase, *BUN* blood urea nitrogen

There was no significant difference in urinalysis values between rats treated with MHEE compared with vehicle controls (Table 5).

**Table 5:** Urinalysis of rats treated with MHEE for 4 weeks

Group	Volume (mL)	Glucose^a^	Bilirubin^a^	Ketone body^a^	Specific gravity	pH	Urobilino-gen^a^
Male rats							
0 mg/kg/day	27 ± 9.9	0/5	0/5	2/5	1.007 ± 0.0027	7.0 ± 0.00	0/5
500 mg/kg/day	22 ± 5.7	0/5	0/5	3/5	1.010 ± 0.0035	6.9 ± 0.22	0/5
1000 mg/kg/day	25 ± 18.0	0/5	0/5	3/5	1.010 ± 0.0035	7.0 ± 0.00	0/5
2000 mg/kg/day	22 ± 11.4	0/5	0/5	4/5	1.012 ± 0.0045	6.8 ± 0.27	0/5
Female rats							
0 mg/kg/day	12 ± 5.7	0/5	0/5	2/5	1.015 ± 0.0061	6.7 ± 0.45	0/5
500 mg/kg/day	11 ± 6.8	0/5	0/5	3/5	1.014 ± 0.0042	6.8 ± 0.27	0/5
1000 mg/kg/day	10 ± 1.8	0/5	0/5	5/5	1.017 ± 0.0027	6.5 ± 0.35	0/5
2000 mg/kg/day	12 ± 6.7	0/5	0/5	4/5	1.015 ± 0.0035	6.6 ± 0.22	0/5

Values are presented as mean ± SD

^a^Number of animals with sign/Total number of animals observed

### Cytotoxicity of MHEE

**Fig. 5:**
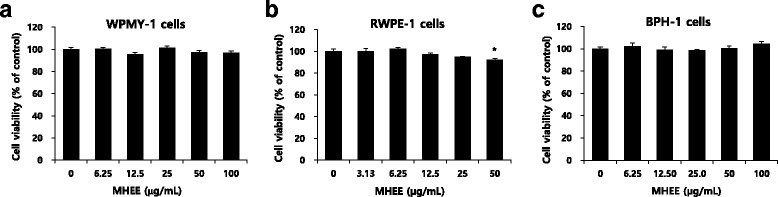
Cytotoxicity of MHEE in prostate cell lines. WPMY-1 (**a**), RWPE-1 (**b**), and BPH-1 (**c**) cells seeded into 96-well plates were incubated with the indicated concentration of MHEE for 24 h. The effect of MHEE on cell viability was evaluated using a CCK-8 assay. Error bars indicate mean values ± SEM of at least three independent experiments. *P < 0.5, compared with vehicle (0 μg/mL)

To determine cytotoxicity of MHEE, the viability of WPMY-1, RWPE**-**1, and BPH**-**1 cells was assessed using a CCK-8 assay after MHEE treatment. The viability of WPMY-1 and BPH-1 cells was barely affected by up to 100 μg/mL of MHEE (Fig. 5a and c). Up to 25 μg/mL of MHEE had no significant effect on viability of RWPE-1 cells, whereas 50 μg/mL of MHEE only slightly inhibited their viability (Fig. 5b). These data suggest that MHEE exhibits no cytotoxic effect in prostate cell lines.

## Discussion and conclusion

Oral administration of MHEE to rats at doses of up to 2000 mg/kg/day for 4 weeks caused no significant changes in clinical signs, body and organ weights, food consumption, necropsy, hematology, serum biochemistry, or urinalysis, regardless of sex. However, there were minor changes in some rats treated with the highest doses of MHEE used in the present study.

Salivation in clinical signs was observed in over half of all rats treated with the highest doses of MHEE. However, it was not accompanied with other pathological changes, including body weight or any gross findings. Moreover, bitter taste, which is found among medicinal plant compound including alkaloids, glycosides, and flavonoids, stimulate rats to secrete saliva [12, 13]. MHEE also includes the compound of flavonoids. Therefore, we regarded the salivation induced by MHEE treatment to be caused only by its bitter taste.

Because the thyroid is small and changes in its weight are caused by factors such as thyroxin and hepatic enzymes, thyroid weight is a relatively less useful indicator of toxicity than the weight of other organs [14]. In this study, administration of MHEE at the highest dose caused increases of thyroid/parathyroid and liver weight. However, changes of thyroid/parathyroid and liver weight by MHEE were not accompanied by gross findings and were just observed in single sex. Therefore, we considered the increase of the thyroid/parathyroid and liver weight after administration of MHEE at the highest dose to be of relatively little toxicological importance.

In terms of hematology, increase of monocyte levels and decrease of basophil levels were observed in rats after administration of the highest doses of MHEE. However, these hematological changes were within the normal physiological range (monocytes: 2.1–3.4 % and basophils: 0.3–1.0 %) [15], and were detected only in male rats, suggesting that it was not considered to be related to MHEE toxicity.

Serum biochemistry is important for monitoring liver function in toxicology studies [16–18]. The highest doses of MHEE caused an increase of GLU, TP, and ALB levels in female rats. Ca concentration, which reflects the status of skeletal mineralization, neuromuscular conduction, blood coagulation, and permeability of sodium and potassium in the body [19], was also increased significantly in female rats after administration of the highest doses of MHEE. However, these changes in serum biochemistry did not exceed the normal range expected for these substances (GLU 113–185 mg/dL, TP 6.1–7.0 g/dL, ALB 3.5–5.1 g/dL, and Ca 9.5–11.0 mg/dL) [15] and were found only in female rats, suggesting that they were not related to MHEE.

In our previous studies, it has been shown that Melandrium firmum methanolic extract effectively inhibits the development of BPH induced by testosterone in a rat model [8]. Therefore, we examined cell viability in vitro to evaluate whether MHEE have the cytotoxicity in prostate cells. Because MHEE had no significant effect on the viability of WPMY-1, RWPE-1, or BPH-1 cells, it may be useful to study the efficacy of MHEE in a rat model of testosterone-induced BPH. The toxicity of MHEE for other cell lines should be evaluated to access further various disease.

In this study, we demonstrated that oral administration of MHEE to rats at doses of up to 2000 mg/kg/day for 4 weeks have no adverse effects. However, further studies including subchronic toxicity and genotoxicity studies might be necessary to determine definitely the oral safety dose of MHEE. Therefore, subchronic toxicity of MHEE should be proceeded on the basis of oral doses of MEHH in subacute toxicity test.

In conclusion, our findings in vivo and in vitro provide information regarding the safety of MHEE. MHEE did not result in any specific adverse effects when administered to Crl:CD Spradgue Dawley rats, regardless of sex, at doses of up to 2000 mg/kg/day for 4 weeks. In addition, MHEE at up to 100 μg/mL showed no significant cytotoxic effects against various prostate cell lines as measured by their viability in vitro. The findings suggest that MHEE in Crl:CD Spradgue Dawley rats is safe as a medicine for at least a month. The subchronic toxicity of MHEE should be assessed further in rat models to establish its safety and toxicity profile.

## Abbreviations

A/G: Albumin/globulin ratio
ALB: Albumin
ALP: Alkaline phosphatase
ALT: Alanine aminotransferase
ANOVA: A one-way analysis of variance
AST: Aspartate aminotransferase
BIL: Bilirubin
BPH: Benign prostatic hyperplasia
BUN: Blood urea nitrogen
CCK-8: Cell counting kit-8
CK: Creatine kinase
CREA: Creatinine
FBS: Fetal bovine serum
GGT: Gamma glutamyl transpeptidase
GLU: Glucose
HCT: Hematocrit
HGC: Hemoglobin concentration
HPLC: High-performance liquid chromatography
IP: Inorganic phosphorus
KET: Ketone bodies
MCH: Mean corpuscular hemoglobin
MCHC: Mean corpuscular hemoglobin concentration
MCV: Mean corpuscular volume
MHEE: Ethanolic extract of Melandrii herba
PL: Phospholipids
PLT: Platelet
RBC: Red blood cell
RET: Reticulocyte
SG: Specific gravity
TBIL: Total bilirubin
TCHO: Total cholesterol
TG: Triglyceride
TP: Total protein
URO: Urobilinogen
WBC: White blood cell
